# The Imbibition, Viability, and Germination of Caper Seeds (*Capparis* *spinosa* L.) in the First Year of Storage

**DOI:** 10.3390/plants11020202

**Published:** 2022-01-13

**Authors:** María Laura Foschi, Mariano Juan, Bernardo Pascual, Nuria Pascual-Seva

**Affiliations:** 1 Departamento Producción Vegetal, Universitat Politècnica de València, 46022 Valencia, Spain; mafos@doctor.upv.es (M.L.F.); mjuan@upv.es (M.J.); bpascual@upv.es (B.P.); 2 Horticulture and Floriculture, Agriculture Faculty, National University of Cuyo, Mendoza M5528AHB, Argentina

**Keywords:** germination curve, germination percentage, seed moisture, tetrazolium

## Abstract

The caper is a shrub that adapts to harsh environments when it is established, but it presents serious difficulties in its propagation, both by cuttings and by seeds. Its seeds have low germination percentages, and germination is a very slow process. Significant increases in germination have been obtained with scarification and with the addition of gibberellic acid (GA_3_) to the substrate, leading to the hypothesis that they have possible physical and physiological dormancy. However, the only way to examine the water-impermeability of the cover is through imbibition analysis. This study analyzes the imbibition, viability, and germination of two seed lots, obtained in different years and evaluated immediately after their collection (FS) and after being stored (7 °C) for one month (DS) and one year (SS). The seed moisture content stabilizes from the fourth day, exceeding in all cases 31% in all three seed states tested (FS, DS and SS). This allows the germination of all viable seeds, only with the addition of GA_3_ to the germination substrate, without the need for scarification, so that caper seeds exclusively appear to present a physiological latency. Germination decreased in storage, even with just one month. With the GA_3_ addition, high germination values were obtained (up to 95% in FS).

## 1. Introduction

The caper (*Capparis spinosa* L.) is a perennial shrub cultivated in the Mediterranean region, which can grow spontaneously in arid or semiarid areas. It has a creeping bearing and can reach a height of up to 0.5 m, having flexuous branches up to 3 m in length and deep roots making it resistant to drought [1,2]. It has solitary axillary flowers and flower buds; the fruit is an ovoid berry containing more than 150 seeds. The seeds are reniform, dark brown, and small with an average maximum Feret diameter of 3.3 mm.

It has a high agricultural potential since it presents a great variety of uses [3,4]. These include highlighting food (for its flower buds and immature fruits, which are usually pickled in brine [1,5]), in the pharmaceutical industry to prevent, among other things, cardiovascular and gastrointestinal diseases [6,7,8,9], and in xero-gardening and landscaping for its ornamental value [2,10], its resistance to drought, and its ability to reduce soil erosion [3,11].

One problem this species presents is the efficiency of propagation, both vegetative and sexual, through seeds. Although the fruits contain many seeds, they have a very low germination percentage. Several authors have reported various studies to improve its germination and try to break the possible physical dormancy with different types of scarification, whether mechanical, chemical, thermal, or biological, [12,13,14,15], as well as being able to break the physiological dormancy with the use of gibberellic acid (GA_3_) and potassium nitrate, as reported by [16,17,18,19]. It has been proven that scarification with sulfuric acid and the addition of GA_3_ have improved the germination percentage [12,20], which has led to the hypothesis that caper seeds can present physical dormancy (due to the impermeability of their cover) and physiological dormancy imposed by the embryo. However, recently, [18] verified that the imbibition takes place through the hilum, and [14] obtained germination percentages up to 99% with only contribution of GA_3_ without scarification, so they hypothesized that the dormancy caused by a waterproof seed coat should not be considered.

The longevity, also referred to as the half-life of the seeds, determined as the time taken for 50% of the seeds to die [21,22,23,24], of a caper seed lot stored at 7 °C and obtained by our research team was around 4 years (3.85 years [20]; 4.15–4.43, for two seed lots [14]). However, both studies recommended a storage period of no longer than two years, because during this period, the viability did not decrease and high germination percentages were obtained. In previous studies [25], our group obtained higher and faster germination in freshly harvested caper seeds compared to that obtained in seeds collected several weeks before performing the germination test.

As Orozco-Segovia [26] stated, the only way to determine if seed coats are water-permeable is to conduct imbibition studies. In this sense, an imbibition test was performed according to [18] using the Between Paper method (BP) [27], reproducing the conditions of the germination test, since in previous studies [14], no significant differences were detected between the moisture contents of the seeds soaked in a 10 cm water column and moistened with the BP method, neither with the use of water nor GA_3_ to moisten the seeds.

The low germination percentages obtained with seeds from commercial lots are known [18]. Thus, the research team is conducting different studies to make the use of these seeds viable and profitable. The objective of this work is to evaluate the effect of the state of the seeds in the imbibition, viability, and germination during the first year of storage, specifically the state of the seeds immediately after the collection and extraction of the fruits (fresh seeds, FS), seeds stored for 30 days (dried seeds, DS), and seeds stored for one year (stored seeds, SS). Given that in previous studies [12,18], the application of GA_3_ to the substrate significantly increased the caper seeds’ germination, the substrate was moistened both with water or a GA_3_ solution in the germination test.

## 2. Materials and Methods

### 2.1. Plant Material

The seeds used in this experiment correspond to two lots of caper seeds produced by adults grown in an experimental plot at the Universitat Politècnica de València (39°29′02.1″ N 0°20′23.9″ W; Valencia, Spain). The harvesting of the seeds in each lot was carried out during the second half of September in 2019 and 2020, one lot for each year. The seeds were classified into three groups according to the drying (or not) and the storage period: fresh seeds (FS), extracted from the fruits, cleaned, and set to germinate immediately without letting them dry; dried seeds stored for 30 days (DS); and dried seeds stored for one year (SS).

The seeds were extracted from ripe fruits collected on the day of their dehiscence and from fruits located in the position before and after it. Then, the seeds were disinfected with sodium hypochlorite for 10 min and rinsed twice in tap water. The mature seeds were selected by rejecting the light seeds through flotation in tap water. Flotation is a common method for separating viable from nonviable seeds, which involves placing the seeds in water so that heavy, sound seeds sink to the bottom and the lightweight and unfilled seeds float to the top [28]. Once the mature seeds were separated, the tests for the FS started. The rest of the seeds were dried in the shade at room temperature (23–25 °C, 20–50% relative humidity) for two weeks, after which they were kept in closed airtight containers at 7 ± 0.5 °C in a domestic refrigerator (Beko, Beko Electronic España, Barcelona, Spain) until the tests were conducted.

### 2.2. Imbibition

The imbibition test was performed according to [18] using the BP method [27], and the seeds were soaked through the paper, which was moistened with pure water (Wasserlab G.R. Type II analytical grade water system; referred to as water). The determinations were made over 8 days to ensure maximum absorption of solution, as indicated by [26]. The BP method was performed with 9 cm diameter Petri dishes and two layers of filter paper, Whatman No 1 [27], at laboratory room conditions (23–25 °C, 20–50% relative humidity). Four replications of ten seeds each per seed status and year of production were considered. The moisture content of the seeds was determined according to the ISTA standards [27] and the water absorption (imbibition) according to the methodology described in [26], for which, the seeds were removed from the Petri dishes hourly during the first day and once every day afterwards. They were blotted with a paper towel, immediately weighed on a precision balance (Sartorius, model B 120S, Barcelona, Spain), and returned to the Petri dish [18]. The calculation of the imbibition is presented as the accumulated percentage of absorbed water, expressed as the increase in fresh weight (%) in each day (i) with respect to the initial weight of the seeds:

Imbibition (%) = 100 ∗ (Fresh weight_i_ − Initial fresh weight)/Initial fresh weight (1)

After the imbibition period, the four samples of each treatment were dried for 48 h at 103 °C in a forced-air oven (Selecta 297; Selecta, Barcelona, Spain) to determine the dry weight. The daily seed moisture content (including the initial seed moisture) was calculated on a fresh mass basis [27]:

Seed moisture (%) = 100 ∗ (Fresh weight_i_ − Dry weight)/Fresh weight_i_ (2)

### 2.3. Viability

The viability and vigor of the seeds were determined by the tetrazolium topographic test according to the International Rules for Seed Analysis [27]. Four replications of fifty seeds each were performed. Seeds were soaked in water at 20 °C for 18 h for preconditioning, after which they were cut longitudinally off at the widest Feret diameter and soaked in a 1% Tetrazolium solution (Tetrazolium Red. 2,3,5-Triphenyltetrazolium chloride; Sigma) at 30 °C for 18 h [29]. Seeds were evaluated with a photomicroscope (U500X Digital Microscope; Cooling Tech, Guangdong, China) with the radicle tip the maximum area of unstained tissue permitted to consider a seed as viable. The viability is the percentage of normal germinable seeds to be expected when the seed lot is germinated under favorable conditions and includes sound (staining proceeds gradually and uniformly from the exposed surfaces inward where changes in the color intensity are gradual without distinct boundaries) and weak but viable (stain greyish red or brighter red than normal) tissues, as reported by [30].

### 2.4. Germination

Germination tests were carried out with the BP method following the International Rules for Seed Testing [27], which consists of using filter paper as a substrate and placing the seeds between two layers of filter paper in Petri dishes of 9 cm in diameter. The samples consisted of 400 seeds (4 replications of 100 seeds). The paper (Whatman No 1) was moistened with two solutions, one of 500 mg·L^−1^ of GA_3_ (Semefil L; Nufarm), and another of water. In both cases, 2 g·L^−1^ of Captan (Captan 50; Bayer) was added to the treatments to avoid contamination with fungi. Petri dishes were placed in a growth chamber (model Zimbueze, Seville, Spain) at 30 ± 1/20 ± 1 °C, 85 ± 1% relative humidity for a photoperiod of 12 h (cold white fluorescent tubes (Philips TL-D 36W/54), providing 81.1 μmol m^−2^ s^−1^) for a maximum of 120 days.

The seeds were considered germinated when the emerged radicle reached a length of 2 mm. The trials were judged as satisfactory when the difference between the maximum and minimum germination percentages of the four replications did not exceed the tolerance established by [27]. For the analysis of the germination curves of each repetition, according to studies carried out previously by [14,30], the model that best fits is that of the logistic function proposed by [31] and has the following expression:

*G* = *A*/1 + e(*β − kt*) (3)

where *G* is the percentage of accumulated germination, *A* is the maximum germination percentage, *t* is the germination period in days, *β* is a parameter of the function concerning the position of the curve relative to the time axis, and *k* is a velocity parameter. Both are used to calculate parameters with biological significance, such as the number of days needed to reach 50% of the final germination percentage (*β*/*k* = *Gt*_50_) and the average relative rate of cumulative germination (*k*/2, day^−1^).

### 2.5. Statistical Analysis

The results were analyzed using analysis of variance (ANOVA) with Statgraphics Centurion 18 software [32]. For the imbibition and viability tests, the analysis was performed as a two-way ANOVA, and a three-way ANOVA was used for the germination test. The differences were considered significant for a probability of *p* ≤ 0.05%. The percentage data were arcsin √x transformed before analysis to accomplish the normality assumption. In this study, the normality distribution was analyzed by verifying the residuals normal distribution [33] by the Shapiro–Wilk test [32]. The separation of means was performed using Fisher’s minimum significant differences test (LSD test) in *p* ≤ 0.05.

## 3. Results and Discussion

### 3.1. Imbibition

In none of the analyzed parameters (Table 1) were there significant differences between the two years of evaluation, 2019 and 2020. The initial moisture of FS was higher (*p* ≤ 0.05) than that of DS and SS, with no differences (*p* ≤ 0.05) between the latter two.

**Figure 1:**
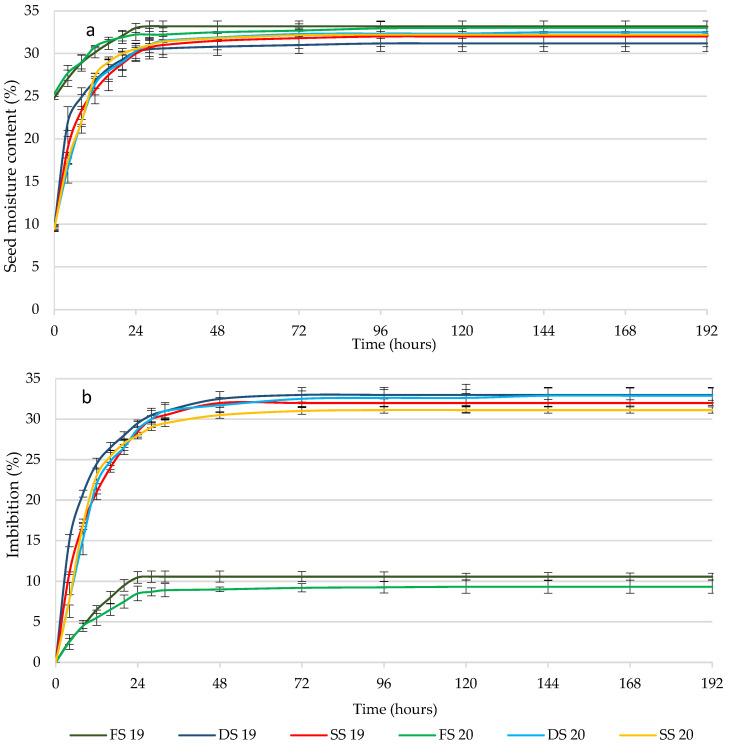
Evolution of the moisture content (**a**) and the accumulated imbibition (**b**) of the seeds (fresh (FS), dried and stored for 30 days (DS), and stored for one year (SS)) during the imbibition process during two years of production: 2019 and 2020. Vertical bars represent the standard error.

These differences in the initial seed moisture (*p* ≤ 0.05) were maintained during the first 24 h, decreasing over time (Figure 1a). There were no differences (*p* ≤ 0.05; Table 1) in the statistical analysis performed at 96 h, in which the moisture content of the three states seeds was about 32%. According to Juan [14], the initial moisture of DS and SS was adequate for its conservation, while the moisture reached in the three seed states was sufficient for efficient germination, which generally ranges between 25 and 50% depending on the species [34,35,36], and particularly for caper seeds as [14,18] stated. The evolution of the imbibition is shown in Figure 1b. The FS presented the lowest imbibition values (*p* ≤ 0.05, Table 1) since at the end of the test, the seeds of the three states reached similar moisture contents (Figure 1a), while the initial moisture of FS was higher (*p* ≤ 0.05) than those of DS and SS (Table 1).

In the seed moisture evolution curves (Figure 1a), the first two phases of the absorption of the three-phase model of water absorption in the germination of the seeds are clearly observed. There is initially a phase of rapid water absorption, followed by a second phase in which it stabilizes and begins the activation of metabolism and the mobilization of nutrients [28,37].

### 3.2. Viability

As Bewley et al. [37] reported, many factors can affect the initial quality of the seeds before storage, particularly the maturity of the seeds at harvest, the conditions during drying, and the handling of the seeds before starting to monitor their viability. The initial viability of the seeds was very high (on average 95.9% for FS) as expected, due to the fruits being harvested at their physiological maturity and the careful extraction, cleaning, and handling of the seeds. There were also no differences (*p* ≤ 0.05) between seed lots (seeds collected in 2019 and 2020).

Viability showed a slight tendency to decrease with drying and storage, but these differences were not significant (Table 2), so it can be indicated that viability was maintained during the first year of storage. The conditions in which seeds are dried and stored greatly affect their deterioration rate and, hence, their ability to survive in storage. Among the factors that can influence the evolution of seed viability, the two most important are its moisture content and the storage temperature [37]. The low moisture content of the seeds can cause their deterioration due to desiccation damage [38]; deterioration is caused by lesions from drying out or a longer time of storage and by the inability to repair these lesions. Unrepaired lesions can delay or avoid the cellular changes needed for the completion of germination, leading to cell dysfunction and death. The spatial relationships between the molecules determine the level of viability of the seeds and their longevity [38,39]. In this case, the seeds were dried in the shade under laboratory conditions, and after drying, the seeds were stored at a low temperature (7 °C), that is, under ideal conditions for drying and storage.

### 3.3. Germination

**Figure 2:**
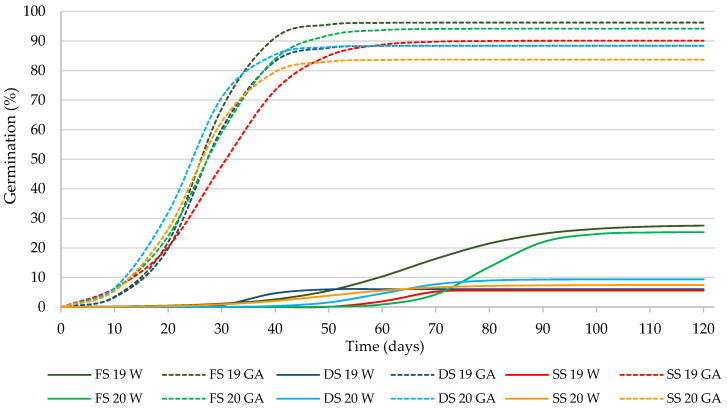
Logistic model adjusted to the germination curves of the seeds (fresh (FS), dried and stored for 30 days (DS), and stored for one year (SS)) for two years of harvesting (2019 and 2020), and with the substrate moistened with distilled water (W) and with a solution of GA_3_.

In all cases, the germination data were adjusted to the logistic function (*p* ≤ 0.01), and the coefficients of determination (R^2^) for the 48 curves of the adjusted germination model presented values greater than 98.4% (data not shown). This indicates that the use of the logistic function is suitable for analyzing the germination of caper seeds (Figure 2), as in previous studies [18,20]. This allowed the use of variable A (instead of G) to analyze the germination percentage of the seeds, as well as the constants β and k, to be able to calculate the biological parameters of germination G*t_50_* and *k*/2, as was used in previous studies on the germination of caper seeds [18,20].

The high percentages of germination obtained with GA_3_ are consistent with those of viability (item 3.2), since the results obtained in the viability and germination tests when properly conducted are generally very close [29]. These germination percentages are also in accordance with those obtained by our team in studies previously conducted with our seeds, in which longevity of around 4 years was estimated (3.85 years, [20]; 4.15–4.43, for two seed lots [14]), in which germination remained nearly constant during an initial period, declining thereafter. The results obtained in this study, considered together with those obtained in the aforementioned works, coincide with those reported by [37], in the sense that the shape of the viability curves are often symmetrically sigmoid once viability begins to decline.

Germination was significantly higher (*p* ≤ 0.01) after moistening the substrate with the GA_3_ solution than with water (on average 90.2% and 13.6%, respectively). In relation to the production year, no significant differences were observed for any of the germination parameters analyzed. The factor that had the greater effect on germination was the solution used to moisten the substrate, which explained 96% of the variability of the data (Table 2). This shows the importance of using gibberellins to obtain high germination percentages in caper seeds.

**Figure 3:**
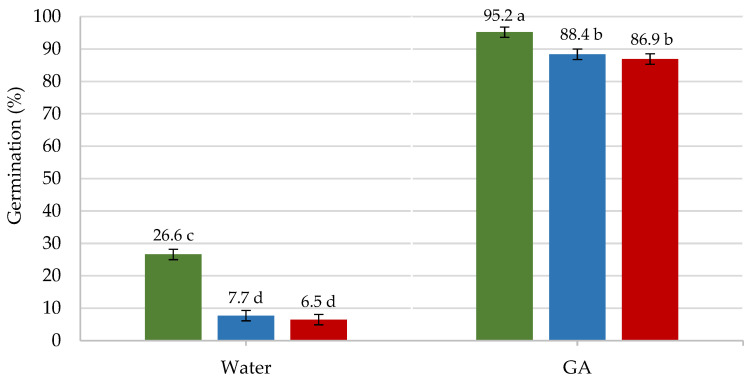
Analysis of the significant interaction between the state of the seeds (fresh (FS) in green, dried and stored for 30 days (DS) in blue, and for one year (SS) in red) and the saturation solution used, water and GA_3_, in the final germination. Different letters in each bar indicate significant differences at *p* ≤ 0.05 using the Fisher’s least significance difference (LSD shown as error bar) test.

From the analysis of the significant interaction (*p* ≤ 0.01; Table 2 and Figure 3), “State of the seeds * Solution to moisten the substrate”, it was found that the FS germinated in greater proportion than those stored, both with water (26.6%) and with the addition of GA_3_ to the substrate (95.2%). The values of final germination with the addition of GA_3_ practically coincided with those of viability, that is, all seeds considered viable germinated. It is worth highlighting the role played by GA_3_ as mentioned above. The high germination percentages obtained with the addition of GA_3_ to the substrate (≥87%) coincided with those obtained in other seed lots of own production obtained in different seasons [14,18]. The high germination percentages maintained during the first year of storage permit the seed distribution and sowing during an entire year, allowing sowing when the weather conditions are right, without requiring the use of such techniques as priming, that present greater or lesser complexity.

Regarding the *Gt_50_*, it was only affected (*p* ≤ 0.01) by the solution used, and according to [18], the GA_3_ reduced the value of *Gt*_50_ (26 d) with respect to water (58 d). Storage for a year slightly (but not significantly) increased the *Gt*_50_ in relation to FS and DS, which agrees with [20]. *k*/2 was not affected (*p* ≤ 0.05) by the storage period of the seeds or the solution used.

## 4. Conclusions

In the germination process, water absorption by seeds increased considerably in the first 24 h, reaching approximately 80% of the total water absorbed. The seed moisture content stabilized after 96 h, reaching a similar value (32%) in the three seed states tested (fresh and after being stored for one month and one year). This allows the germination of all viable seeds, only with the addition of GA_3_ to the germination substrate, without the need for scarification. The use of gibberellins was essential to obtain germination percentages close to those of the viability of the seed lot. The viability of the seeds decreased slightly with drying and storage, although without statistical significance. The germination percentage decreased significantly with drying and initial storage, remaining similar in value after storage for one month and one year. From the abovementioned, it can be concluded that caper seeds do not present physical dormancy caused by the impermeability of the seed coat and that the low germination could be due to a non-deep physiological dormancy. The information obtained in this study may be of great interest for seed producer companies and nurseries to obtain viable and profitable caper propagation. Further studies will be carried out with seeds in their first year of storage applying GA_3_, using seedling trays and pots, both in the greenhouse and in the field to transfer this knowledge to the process of obtaining plants in the nursery.

## Figures and Tables

**Table 1:** Effect of the state of seeds (fresh (FS), stored for 30 days (DS), and stored for one year (SS)) on its moisture (M; %) and its accumulated imbibition (I; %) after 0, 12, 24, and 96 h in two years of production.

	0 h	12 h		24 h		96 h	
	M	I	M	I	M	I	M
State of the seed (S)							
FS	25.1 a	5.8 b	30.5 a	9.3 b	32.7 a	9.9 c	33.1
DS	9.8 b	22.9 a	26.1 b	28.9 a	29.6 b	33.0 a	31.8
SS	9.5 b	21.5 b	26.2 b	28.7 a	30.3 b	31.5 b	32.2
Year (Y)							
2019	14.7	16.6	26.9	22.9	30.7	25.3	32.2
2020	14.8	16.9	28.3	21.6	31.0	24.3	32.5
Analysis of Variance							
Source (degrees of freedom)	% Sum of squares						
S (2)	99.8 **	92.0 **	54.8 **	94.07 **	47.5 **	98.9 **	13.4 NS
Y (1)	0.0 NS	0.0 NS	6.1 NS	0.5 NS	0.8 NS	0.2 NS	1.1 NS
S × Y (2)	0.0 NS	1.2 NS	3.4 NS	0.2 NS	6.5 NS	0.0 NS	4.1 NS
Residuals (18)	0.1	6.8	35.7	4.5	45.2	0.9	81.3
Standard deviation	0.3	2.4	1.9	2.3	1.5	1.2	1.6

Mean values followed by different lower-case letters in each column indicate significant differences at *p* ≤ 0.05 using the Fisher’s least significance difference (LSD) test. NS indicates not significant differences. ** Indicates significant differences at *p* ≤ 0.01.

**Table 2:** Effects of the state of the seeds (fresh (FS), dried and stored for 30 days (DS), and stored for one year (SS)), of the year of production and of the saturation solution used, on viability (V; %), accumulated germination (*G*; %), final germination (*A*; %), number of days needed to reach 50% of *A* (*Gt*_50_), and the average relative rate of accumulated germination (*k*/2, day^−1^).

	*V*	*G*	*A*	*Gt*_50_	*k/2*
State of the seed (S)					
FS	95.9	60.9 a	60.9 a	40.4	0.081
DS	90.1	48.4 b	48.1 b	38.8	0.112
SS	89.2	47.1 b	46.7 b	44.5	0.099
Year (Y)					
2019	92.5	52.5	52.4	39.4	0.108
2020	90.4	51.7	51.4	44.4	0.088
Saturation solution (Sol)					
Water	----	13.6 b	13.6 b	58.0 a	0.104
GA_3_	----	90.7 a	90.2 a	25.8 b	0.091
Analysis of Variance					
Sources (degrees of freedom)	% Sum of squares				
S (2/2)	36.6 NS	2.5 **	2.7 **	1.7 NS	4.8 NS
Y (1/1)	4.0 NS	0.0 NS	0.0 NS	1.4 NS	2.8 NS
Sol (0/1)	----	96.4 **	96.1 **	58.2 **	1.2 NS
S × Y (2/2)	0.3 NS	0.0 NS	0.1 NS	4.7 NS	4.0 NS
S × Sol (0/2)	----	0.4 **	0.5 **	1.0 NS	5.6 NS
Y × Sol (0/1)	----	0.0 NS	0.1 NS	3.0 NS	2.8 NS
S × Y × Sol (0/2)	----	0.0 NS	0.1 NS	2.7 NS	11.8 NS
Residuals (18/36)	59.0	0.5	0.5	27.3	67.1
Standard deviation	4.6	3.2	3.2	12.7	0.1

Mean values followed by different lower-case letters in each column indicate significant differences at *p* ≤ 0.05 using the Fisher’s least significance difference (LSD) test. NS indicates not significant differences. ** Indicates significant differences at *p* ≤ 0.01.
